# Pytaxon: A Python software for resolving and correcting taxonomic names in biodiversity data

**DOI:** 10.3897/BDJ.13.e138257

**Published:** 2025-01-08

**Authors:** Marco A. Proença Neto, Marcos P. A. De Sousa

**Affiliations:** 1 Centro Universitário do Estado do Pará, Belém, Brazil Centro Universitário do Estado do Pará Belém Brazil; 2 Laboratório de Computação Avançada para Biodiversidade (COMBIO). Museu Paraense Emílio Goeldi, Belém, Brazil Laboratório de Computação Avançada para Biodiversidade (COMBIO). Museu Paraense Emílio Goeldi Belém Brazil

**Keywords:** biodiversity informatics, taxonomy, scientific names, species name matching, data cleaning

## Abstract

**Background:**

The standardisation and correction of taxonomic names in large biodiversity databases remain persistent challenges for researchers, as errors in species names can compromise ecological analyses, land-use planning and conservation efforts, particularly when inaccurate data are shared on global biodiversity portals.

**New information:**

We present pytaxon, a Python software designed to resolve and correct taxonomic names in biodiversity data by leveraging the Global Names Verifier (GNV) API and employing fuzzy matching techniques to suggest corrections for discrepancies and nomenclatural inconsistencies. The pytaxon offers both a Command Line Interface (CLI) and a Graphical User Interface (GUI), ensuring accessibility to users with different levels of computing expertise. Tests on spreadsheets derived from datasets published in the Global Biodiversity Information Facility (GBIF) demonstrated its effectiveness in identifying and resolving taxonomic errors. By mitigating the propagation of inaccuracies from researchers' datasets to global biodiversity databases, pytaxon supports more reliable conservation decisions and robust scientific investigations. Its contributions enhance data integrity and promote informed biodiversity management in a rapidly evolving global environment.

## Introduction

The scientific names of species and the taxonomic groups to which they belong are fundamental for the organisation and storage of the vast range of information on global biodiversity, functioning as crucial identifiers in this context (Grenié et al. 2022). Assigning a species identification to an organism is a fundamental activity in a wide range of disciplines, including ecology, land-use planning and conservation (Tyrrell 2019). Incorrect, ambiguous or synonymous taxon names present a fundamental problem for the study of comparative biology and directly affect the analysis of species occurrence data needed in ecological, biogeographical and fauna and flora conservation applications (Meyer et al. 2016, Thomson et al. 2021).

Traditionally, species data are often recorded in spreadsheets due to their ability to make the organisation and storage of information more flexible. These spreadsheets, frequently used by researchers, are a primary source for biodiversity databases (Güntsch et al. 2014, Bernardo et al. 2015, Silva et al. 2017, da Costa Prudente et al. 2019, da Silva et al. 2019). However, spreadsheets with large volumes of data are vulnerable to errors and difficult to correct (Broman and Woo 2018) and ensuring high quality species occurrence data in spreadsheets is a challenge (Foley 2010, Brown 2013, Magalhaes 2019). When incorrect data are included in a biological collections database, it can compromise the quality and reliability of scientific research (Moudrý and Devillers 2020). If such information is shared on international biodiversity information portals, the impact of erroneous data could expand globally (Saran et al. 2022).

Although renowned portals such as the Global Biodiversity Information Facility (GBIF; http://www.gbif.org) and the Atlas of Living Australia (ALA; https://www.ala.org.au) allow the import of spreadsheets with biodiversity data and have the ability to identify potentially incorrect or incomplete entries, corrections are not made automatically. It is the responsibility of the data provider to manually correct each identified error (Mesibov 2018). This correction process can become exhaustive when dealing with numerous spreadsheets, as each one needs to be individually reviewed.

Furthermore, there are online platforms that permit the search and resolution of taxonomic names in certain cases, such as the Global Names Verifier (GNV; https://verifier.globalnames.org) and the Taxonomic Name Resolution Service (TNRS; https://tnrs.biendata.org). However, these platforms do not directly interface with spreadsheets and web-based queries are limited to a certain amount of scientific name entries. Ideally, researchers should have at their disposal a free tool that can interact with spreadsheets to validate taxonomic names in a simple and automated way, thereby facilitating the research process.

Currently, there is a variety of R packages available that assist in the process of standardising taxon names, including Taxadb (Norman et al. 2020), WorldFlora (Maitner et al. 2017, Kindt 2020) and U.Taxonstand (Zhang and Qian 2023). However, many researchers encounter difficulties in acquiring programming skills during their academic training (Emery et al. 2021), which can limit the adoption of these tools. This challenge underscores the need for user-friendly graphical interfaces and accessible methodologies to broaden the utilisation of such resources, as elaborated in the subsequent paragraph.

In many studies involving data analysis in biology, the prevalence of software operating with Command Line Interfaces (CLI) is evident, but this becomes a challenge in terms of accessibility, particularly for users with limited computing experience, as noted by Joppich and Zimmer (2019) and Chen et al. (2020). Graphical User Interfaces (GUIs) have emerged as a more convenient alternative, reducing errors in parameter settings and the overall usage burden. While a GUI does not inherently ensure a more user-friendly application, it does facilitate access and operation of the software, making it more accessible and less intimidating for users (Anslan et al. 2017).

To optimise and facilitate the review of taxonomic names in researchers’ spreadsheets, we present pytaxon, a Python-based tool developed to identify and correct errors in species names within spreadsheets. The tool leverages the GNV API to compare user-provided data against trusted taxonomic name databases (https://verifier.globalnames.org/data_sources). Notably, pytaxon offers both a Command Line Interface (CLI) for automation and a Graphical User Interface (GUI) designed for users unfamiliar with programming, ensuring accessibility and usability for a broad audience.

## Project description

### Title

pytaxon

### Design description

Pytaxon is developed in Python and designed to facilitate the validation and correction of taxonomic data in biodiversity spreadsheets. It utilises the Pandas library (Betancourt and Chen 2019) to manipulate and analyse CSV files and Excel spreadsheets, while integrating the GNV API through the requests library to access a comprehensive taxonomic database. The Fuzzy Matching functionality provided by the GNV API is leveraged by pytaxon to suggest corrections for naming discrepancies. Fuzzy Matching is a technique that identifies similarities between taxonomic names by calculating a similarity score to detect and correct typographical errors, improving taxonomic consistency and harmonisation in biodiversity datasets (Grenié et al. 2022). Additionally, the software uses the argparse library (https://pypi.org/project/argparse) for command-line operations to manage input parameters and arguments, making it a versatile tool for users familiar with Python pipelines.

The graphical user interface (GUI) is developed using Tkinter (https://docs.python.org/pt-br/3/library/tkinter.html), providing an interactive platform for users with minimal programming experience. It includes features for visualising data discrepancies and correcting taxonomic errors directly within the interface. Matplotlib is integrated for detailed visual data representation, enhancing user interaction. Pytaxon supports two ways of working: a command-line interface (CLI) for automation and a GUI for ease of use, ensuring accessibility to a broader range of users. The software is open-source and available on GitHub (https://github.com/pytaxon/pytaxon-cli) and PyPI (https://pypi.org/project/pytaxon), where the installation instructions and usage details can be found. This accessibility makes it easy to integrate, modify or use pytaxon for managing and correcting taxonomic data in large biodiversity datasets.

## Web location (URIs)

Homepage: https://doi.org/10.5281/zenodo.14457929

## Technical specification

Programming language: Python 3.10

Operational system: Windows, Linux

## Usage licence

### Usage licence

Other

### IP rights notes

MIT Licence

## Implementation

### Implements specification

**Figure 1.**
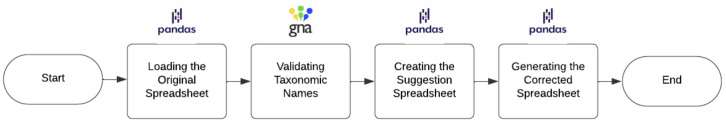
Pytaxon’s workflow.

Below are the steps of pytaxon’s workflow explained in detail; its visual workflow may be viewed at Fig. 1.

**Loading the Original Spreadsheet**

Loading the original spreadsheet is the first step in pytaxon’s workflow, whether using the GUI or CLI. In the CLI, the command -*os* or --*original_spreadsheet* is used to provide the spreadsheet to be analysed, which must be in CSV or Excel format for compatibility. The command -*r* or --*columns* specifies the columns to be read, typically including "Kingdom", "Phylum", "Class", "Order", "Family", "Genus", "Species" and "ScientificName". Users can skip unnecessary columns by using the placeholder x (e.g. -r kingdom, phylum, class, order, family, x, x, scientificName), allowing flexibility in selecting relevant fields.

The -*si* or --*source_id* parameter allows the user to select the database which the programme will use to compare taxonomic names. The IDs for the supported data sources are: 1 for Catalog of Life (COL), 4 for National Center of Biotechnology Information (NCBI), 11 for Global Biodiversity Information Facility (GBIF) and 180 for iNaturalist. Additionally, the -*ss* or --*suggested_spreadsheet* command generates a spreadsheet containing suggested corrections for taxonomic name errors, helping users review and validate discrepancies efficiently.

It is important to clarify that pytaxon focuses on validating and correcting taxonomic names within the dataset, rather than analysing species occurrence data. The frequency of taxonomic names in the dataset is used to identify discrepancies and suggest corrections.

**Figure 2.**
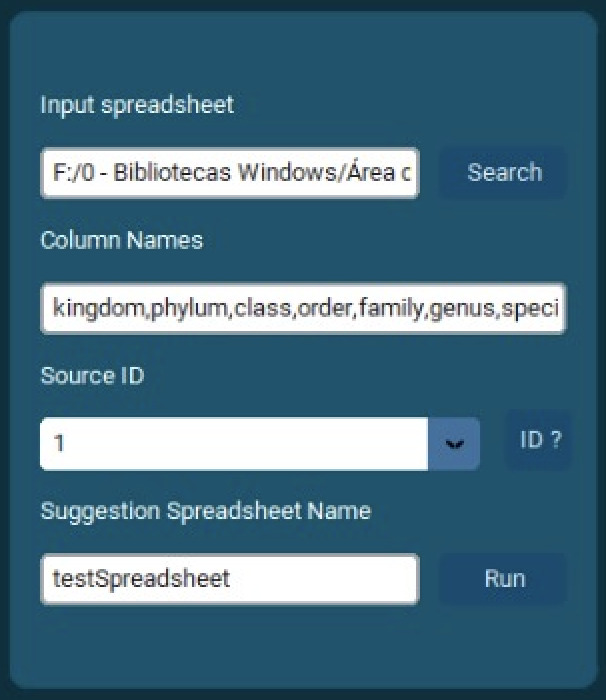
The pytaxon GUI displaying spreadsheet input fields and parameter selection options.

For users who prefer a graphical interface, pytaxon also provides a GUI option for data entry, as shown in the attached Fig. 2. In the GUI, users can easily upload their input spreadsheet by selecting the file through a browse button. They can define the relevant column names directly in the interface, specify the source database ID from a dropdown menu and input a name for the suggestion spreadsheet. The GUI simplifies the process by providing an intuitive interface for data entry and validation.

**Validating Taxonomic Names**

**Figure 3.**
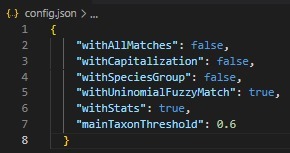
Configuration of the config.json file used in pytaxon.

Taxonomic name validation in pytaxon is achieved through integration with the Global Names Verifier (GNV) API (https://verifier.globalnames.org/api). This process begins with Exact Matching, where names are parsed to exclude authorship details and directly compared to entries in the GNV database. If no exact match is found, the API applies Fuzzy Matching, which corrects minor errors such as misspellings or formatting inconsistencies. These features ensure accurate name validation, even in datasets with imperfections, improving the reliability of biodiversity data. Fig. 3 shows the configuration file (config.json) that defines key parameters for the validation process.

**Figure 4.**
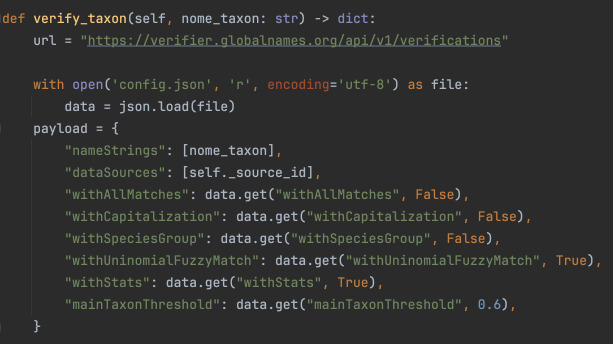
The verify_taxon function that loads the config.json file in pytaxon.

The verify_taxon function, shown in Fig. 4, dynamically loads the config.json file to retrieve and apply the specified parameters. These include withAllMatches, which limits results to the best match to avoid ambiguity and withCapitalization, ensuring case-insensitive comparisons. The parameter withSpeciesGroup focuses the validation on species-level matches, while withUninomialFuzzyMatch activates fuzzy matching for genus-level names, enabling detection and correction of minor errors. The threshold for match acceptance is controlled by mainTaxonThreshold, balancing accuracy and tolerance for variations. Confidence metrics are included in the API response when withStats is enabled, helping to prioritise suggested corrections.

Adjusting the values in config.json allows users to fine-tune the validation process according to their specific needs. For example, increasing mainTaxonThreshold improves precision, while enabling withAllMatches provides broader results. This flexibility, as illustrated in the attached figures, ensures that pytaxon is a customisable tool for standardising and validating taxonomic data in biodiversity datasets.

To address the challenges posed by the lack of standardised outputs from biodiversity database APIs, pytaxon processes and harmonises results from the four databases it currently supports: Catalog of Life, GBIF, NCBI and iNaturalist. This standardisation ensures consistency across outputs, enabling seamless validation and correction of taxonomic names and reducing potential errors arising from incompatible formats. By unifying these diverse outputs into a coherent format, pytaxon simplifies downstream analyses and enhances data reliability. In the future, we plan to extend support to additional databases, further increasing the software's versatility and applicability in biodiversity informatics.

**Creating the Suggestion Spreadsheet**

Based on the results of the Fuzzy Matching process provided by the GNV, pytaxon creates a suggestion spreadsheet with a name specified using the -*ss* or --*suggested_spreadsheet* command. This spreadsheet contains the rows with detected errors, the taxonomic rank (type), the presumably incorrect taxonomic names, the suggested corrections, the taxon source from the selected database and a "Change" column with options “*yes*” or “*no*” for the researcher to validate the corrections. This spreadsheet acts as a detailed report, highlighting areas that need attention and offering a clear and organised approach for rectifying inaccuracies in the dataset.

**Generating the Corrected Spreadsheet**

The final step in pytaxon’s workflow is generating a corrected spreadsheet by applying the validated corrections from the suggested spreadsheet. This process updates the original dataset by replacing erroneous taxonomic names with accurate, user-validated ones. The corrected spreadsheet ensures data accuracy and standardisation, completing the workflow with a dataset of greater reliability.

## Additional information

### Results and conclusion

To demonstrate the advantages and flexibility of the pytaxon software package, we executed both CLI and GUI examples, highlighting its typical applications and core features through queries to analyse a given dataset and retrieve probable corrections. For these simulations, we inserted some errors into Uropygi dataset, which is openly available on the Zenodo platform (Sousa and Proença 2024), changing the Genus type “Thelyphonelus” to “Thelyphoneluss”, the Order type “Uropygi” to “Urophygi” and the Kingdom type “Animalia” to “Aanimalia”, then pytaxon was installed as a package from PyPI and its CLI commands were executed directly from the terminal with relevant queries as arguments, while the GUI version allowed for interactive engagement through its buttons and text blocks. This dual approach showcased the ease of use and comprehensive functionality of pytaxon in managing and correcting taxonomic data.

**Command Line Interface Example**

**Figure 5.**
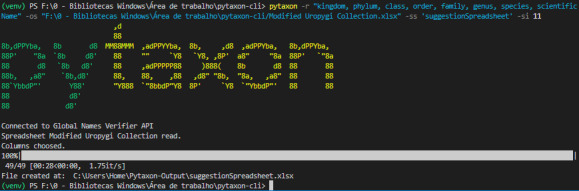
The pytaxon CLI running on the Visual Studio Code terminal (Powershell) with the Uropygi dataset with inserted errors.

The command "*pytaxon -r 'kingdom, phylum, class, order, family, genus, species, scientificName' -os '<original spreadsheet path>/Modified Uropygi Collection.xlsx' -ss 'suggestionSpreadsheet' -si 11*" was used to begin the pytaxon analysing process, as shown in Fig. 5:

To initiate the pytaxon analysis, a dataset containing taxonomic information must be loaded using the appropriate CLI command. For example, the following command specifies the input spreadsheet, the columns to be analysed, the name of the suggestion spreadsheet and the source id:

*pytaxon -os '<original spreadsheet path>/Modified Uropygi Collection.xlsx' -r 'kingdom, phylum, class, order, family, genus, species, scientificName' -ss 'suggestionSpreadsheet' -si 11*

**Figure 6.**
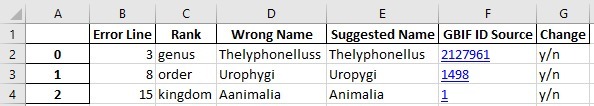
The suggestion spreadsheet identifying errors and providing corrections.

This command specifies the GBIF database for validation (-si 11), though users can select from other supported options such as the Catalog of Life, NCBI or iNaturalist. Once connected to the Global Names Verifier (GNV) API, the programme validates taxonomic names using exact matching to identify precise matches and Fuzzy Matching to address minor errors like misspellings. The generated suggestion spreadsheet, as illustrated in Fig. 6, highlights problematic entries and provides recommended corrections. A key feature of this spreadsheet is the "ID Source" column, which links each taxonomic name to its unique identifier in the reference database, ensuring traceability and reliability of the corrections. Additionally, the spreadsheet includes a "Change" column, allowing researchers to interactively review and decide whether to accept or reject each suggested correction, ensuring flexibility and control over the validation process.

After reviewing and validating the corrections in the suggestion spreadsheet, the user applies the changes to generate a cleaned dataset. This is achieved with the following command:

*pytaxon -os '<original spreadsheet path>/Modified Uropygi Collection.xlsx' -ss '<suggestion spreadsheet path>/suggestionSpreadsheet.xlsx' -o 'correctedSpreadsheet*'

This step creates a new corrected spreadsheet by replacing erroneous names with validated entries, ensuring a standardised and accurate dataset. By automating these processes, pytaxon streamlines the identification and correction of taxonomic errors, enhancing the quality and reliability of biodiversity data.

**Graphical User Interface Example**

**Figure 7.**
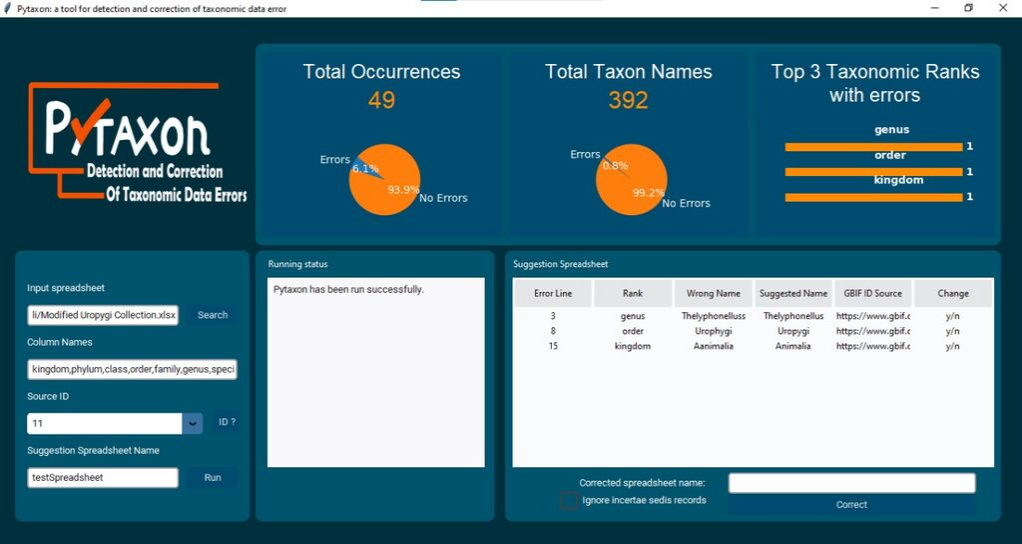
The pytaxon GUI application running with the Uropygi dataset with inserted errors.

The pytaxon graphical user interface (GUI), as shown in Fig. 7, provides a streamlined platform for validating and correcting taxonomic datasets. Its design is structured into functional components, each playing a vital role in the workflow. At the top of the interface, summary panels provide a comprehensive overview of the dataset. The "Total Occurrences" panel displays the overall count of processed records, while the "Total Taxon Names" panel highlights the unique taxonomic names included in the dataset. The "Top 3 Taxonomic Ranks with Errors" panel focuses on the taxonomic levels most prone to discrepancies, helping users identify key areas for review.

The left-hand section of the interface is dedicated to data input and configuration. Here, users can upload their datasets via the "Input Spreadsheet" field, define the taxonomic columns to analyse, select the desired reference database (e.g. GBIF Backbone Taxonomy) and assign a name for the suggestion spreadsheet. Once these parameters are set, the "Run" button starts the analysis process, seamlessly transitioning to the execution phase.

As the analysis runs, the central "Running Status" panel dynamically updates to display progress and alert users to any issues encountered. This real-time feedback ensures users can monitor the process efficiently. On the right side, the "Suggestion Spreadsheet" section lists the detected errors, along with suggested corrections, in an interactive format. Users can review the discrepancies and directly accept or reject each correction within the interface, maintaining control over the final output.

With its integration of configurable input options, interactive error correction tools and live progress monitoring, the pytaxon GUI offers a robust and user-friendly environment for improving the quality of taxonomic datasets. This intuitive design ensures accessibility for users with varying levels of technical expertise, while maximising efficiency in the validation process.

**Unit Testing in pytaxon**

Developing unit tests for the pytaxon software was a important step in ensuring its robustness and reliability. The test suite, named "Test pytaxon" (De Sousa and Proença 2024) was implemented using python's unittest framework (https://docs.python.org/3/library/unittest.html). These tests evaluate critical functionalities, including taxonomic name validation, input file handling and the creation of corrected spreadsheets. This approach ensures that pytaxon performs reliably in both standard and edge-case scenarios.

The tests included specific scenarios to assess compatibility with various input formats, such as CSV and Excel files. Additionally, edge cases, such as empty files, blank lines, and missing columns, were simulated to verify the software’s ability to effectively manage incomplete or inconsistent inputs. Clear error messages and integrated solutions were added to enhance user experience and confidence.

**Comparing Pytaxon to Other Tools**

The pytaxon stands out from other widely used tools, such as the Taxonomic Name Resolution Service (TNRS), Taxadb, WorldFlora and U.Taxonstand, due to its accessibility, flexibility and comprehensiveness. The TNRS is designed exclusively for the standardiation of plant taxonomic names and relies on specific databases like World Flora Online (WFO) and the World Checklist of Vascular Plants (WCVP). In contrast, pytaxon adopts a broader approach, enabling the validation of taxonomic names across a wide range of biological groups. It utilises diverse taxonomic sources, including the Catalog of Life, GBIF Backbone Taxonomy, iNaturalist and the National Center for Biotechnology Information (NCBI).

Another key advantage of pytaxon is its user-friendly interface. While tools like Taxadb and U.Taxonstand require programming skills, pytaxon provides both a Command Line Interface (CLI) and a Graphical User Interface (GUI), making it accessible to researchers with different levels of familiarity in computing. This integration allows for the direct manipulation of spreadsheets in CSV and Excel formats, enhancing efficiency in the processing and validation of taxonomic data.

**Conclusions**

The pytaxon provides an innovative solution to the persistent challenge of standardising and correcting taxonomic names in biodiversity data. By seamlessly integrating a graphical user interface with command-line functionality, the software is accessible to users with diverse technical skills. Leveraging exact and Fuzzy Matching techniques in conjunction with the GNV API, it efficiently detects and resolves taxonomic discrepancies in large datasets. This capability ensures data accuracy and streamlines the validation process for spreadsheets commonly used in biodiversity research. By enhancing the quality of taxonomic data, pytaxon contributes to more reliable ecological analyses, informed conservation planning and robust scientific investigations.
